# Agreement between preload reserve measured by impedance cardiography and echocardiography during pregnancy

**DOI:** 10.1007/s00404-018-4773-x

**Published:** 2018-04-05

**Authors:** Huan Liang, Åse Vårtun, Ganesh Acharya

**Affiliations:** 1 Division of Obstetrics and Gynecology, Department of Clinical Science, Intervention and Technology, Karolinska Institutet, Stockholm, Sweden; 2 Department of Obstetrics, Obstetrics and Gynecology Hospital of Fudan University, Shanghai, China; 3 Women’s Health and Perinatology Research Group, Department of Clinical Medicine, UiT-The Arctic University of Norway, Tromsø, Norway; 4 Center for Fetal Medicine, Karolinska University Hospital, Stockholm, Sweden

**Keywords:** Preload reserve, Impedance cardiography, Echocardiography, Pregnancy

## Abstract

**Purpose:**

Accurate assessment of cardiac function is important during pregnancy. Echocardiography and impedance cardiography (ICG) are commonly used noninvasive methods to measure stroke volume (SV) and cardiac output (CO). The difference in stroke volume (ΔSV) or cardiac output (ΔCO) measured at baseline and after passive leg raising (PLR) is a measure of preload reserve that predicts volume responsiveness. However, the agreement between these two methods in measuring preload reserve during pregnancy is unclear. The aim of our study was to investigate the correlation and the agreement between Doppler echocardiography and ICG in assessing preload reserve in pregnant women.

**Methods:**

In this prospective observational cross-sectional study, preload reserve was assessed by measuring the SV and CO during baseline and 90 s after PLR simultaneously by Doppler echocardiography and ICG in healthy pregnant women during the second and third trimesters. Bland–Altman analysis was used to determine the agreement between the two methods. Bias was calculated as the mean difference between two methods and precision as 1.96 SD of the difference.

**Results:**

A total of 53 pregnant women were included. We found a statistically significant correlation between ΔSV (*R* = 0.56, *p* < 0.0001) and ΔCO (*R* = 0.39, *p* = 0.004) measured by ICG and Doppler echocardiography. The mean bias for ΔSV was 2.52 ml, with a precision of 18.19 ml. The mean bias for ΔCO was 0.21 l/min, with a precision of 1.51 l/min.

**Conclusion:**

There was a good agreement and a statistically significant correlation between ICG and Doppler echocardiography for measuring preload reserve.

## Introduction

Cardiac disease complicates more than 1% of all pregnancies, and cardio-vascular complications are one of the major causes of indirect maternal death accounting for 20% of all cases [1]. Today, many women with surgically corrected congenital heart disease choose to get pregnant. Acquired rheumatic heart disease also remains a problem among pregnant women in developing countries [2]. Profound changes in cardio-vascular function occur during pregnancy, including cardiac output, heart rate, and left ventricular stroke work index. [3–6]. Especially, cardiac output increases dramatically during pregnancy [7, 8] and has a significant pathophysiological impact on the natural history, diagnosis, management, and prognosis of pregnancy complications [9–11]. Therefore, accurate measurement of cardiac output is important during pregnancy. The assessment of maternal hemodynamics may also be useful in the perioperative management of pregnancies that are at risk of volume overload and cardiac failure, such as women with twin pregnancies [12] and women with severe preeclampsia. [10].

Recently, more emphasis has been put on the dynamic assessment of cardiovascular function (functional hemodynamics) rather than on the static measurements [12–17]. Passive leg raising (PLR), a maneuver that consists of passively lifting the lower limbs from the horizontal plane up to 45°, has been used as an endogenous fluid challenge test as it leads to a certain amount of blood volume being auto-transfused into the central circulation, which subsequently can be expected to increase the cardiac output by Frank–Starling mechanism [18]. The change of cardiac output after PLR can predict preload reserve and volume responsiveness [19], which is an important parameter that needs to be considered during fluid therapy, blood transfusion, antihypertensive treatment, etc.

Doppler echocardiography and impedance cardiography (ICG) are two common noninvasive methods used to measure cardiac output, and several studies have validated the use of both these methods against other gold standard methods to measure cardiac output [20–22]. However, the agreement between these two methods of measuring preload reserve during pregnancy is unclear.

Thus, the aim of our study was to investigate the correlation and the agreement between Doppler echocardiography and ICG in assessing preload reserve in pregnant women.

## Materials and methods

### Study design and population

This was a prospective observational cross-sectional study. The participants recruited to the study were healthy pregnant women attending the antenatal clinic of the University Hospital of North Norway, Tromsø, Norway, in the second or third trimesters of pregnancy for routine antenatal checkups. Eligible women were informed about the study, and those who consented to participate were recruited consecutively.

### Inclusion and exclusion criteria

Healthy women older than 18 years with uncomplicated singleton pregnancy were included at 17–41 weeks of gestation. Exclusion criteria were: any pre-existing medical condition that may have an effect on the course and outcome of pregnancy, such as diabetes mellitus and heart disease, and obstetric complications, such as hypertensive pregnancy disorders and gestational diabetes, in the current pregnancy or any clinical suspicion of heart failure, and the presence of fetal aneuploidy or any major fetal or placental abnormality detected on ultrasound examination.

### Medical history and anthropometry

A thorough medical history was obtained, and the women’s age and parity were recorded. A general clinical examination was performed to exclude any obvious signs of heart failure. Women’s weight was measured using an electronic scale (Soehnle, Leifheit AG, Nassau, Germany) and the height using an altimeter (Charder Electronic Co, Taichung City, Taiwan). The body mass index (BMI) was calculated as weight (kg)/height (m)^2^.

### Assessment of maternal hemodynamics

**Fig. 1:**
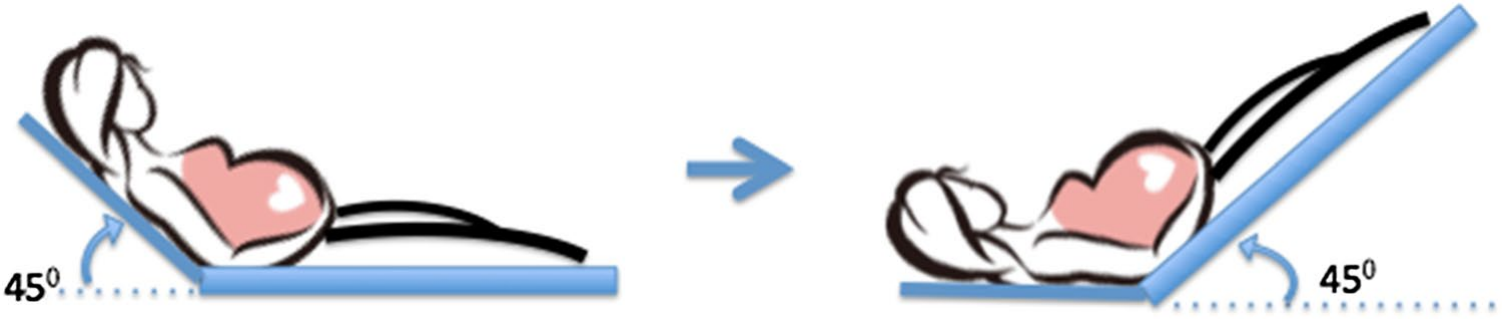
The technique of passive leg raising used to assess preload reserve

Blood pressure (BP) was measured using an appropriate size sphygmomanometer cuff placed on the upper arm with women resting in a semi-recumbent position. The mean arterial pressure (MAP) was calculated as diastolic BP + 1/3 (systolic BP − diastolic BP). Stroke volume (SV) and cardiac output (CO) were measured simultaneously using two different methods (Doppler echocardiography and ICG) at baseline and 90 s after PLR. Using Doppler echocardiography, the SV was calculated as: time velocity integral (VTI) of the aortic blood flow velocity multiplied by the cross-sectional area of the aortic annulus [23], whereas with ICG, the SV was calculated based on changes in thoracic electrical bio-impedance signals as aortic blood flow velocity and blood volume change with each heartbeat using the modified Kubicek–Sramek–Bernstein equation [24]. The CO was calculated as the product of SV and heart rate. Baseline measurements were performed with the participant in a 45° supine semi-recumbent position after at least 10 min of rest on an electronically pivotable bed that allows changing the maternal position without any active movement by the woman herself [16]. Then, the participant’s legs were raised up to a 45° angle while the head and the chest were placed to a supine position on the bed. Ninety seconds after leg raising, the Doppler echocardiography and the ICG were performed again (Fig. 1). The participants were requested not to move and remain quiet during the whole measurement procedure.

### Doppler echocardiography

Doppler velocity waveform of the left ventricular outflow was obtained by a single operator with formal training in echocardiography and several years of experience in perinatal cardiology (GA) using a 2 MHz continuous wave Doppler pencil probe attached to a VIVID 7 ultrasound system (GE Vingmed Ultrasound, Horton, Norway). The pencil probe was placed at the suprasternal notch and aligning the Doppler insolation angle with the direction of aortic blood flow. Doppler blood flow velocity waveforms from at least three cardiac cycles were recorded for off-line analysis. The blood flow velocity waveforms were traced to obtain maternal heat rate (HR_echo_) and the velocity time integral (VTI) of the aortic flow velocity using the software of the ultrasound machine. The aortic annulus diameter was calculated using the Nidorf equation as: 0.01 × height (cm) + 0.25, as aortic annulus diameter is known to have an extremely good correlation with the individuals height [25], and the aortic annulus area was calculated as: 3.14 × (aortic annulus diameter/2)^2^. The SV_echo_ was calculated as the product of aortic annulus area and VTI of the aortic blood flow velocity. The CO_echo_ = SV_echo_ × HR_echo_.

### Impedance cardiography

The ICG was performed by another single experienced operator (ÅV) as described previously [16] using a standard ICG machine (Philips Medical Systems, Androver, MA, USA) [23]. During the measurements, stroke volume (SV_ICG_), heart rate (HR_ICG_), and cardiac output (CO_ICG_) were continuously displayed on the ICG screen. A screenshot of the ICG machine was printed simultaneously when the aortic blood flow velocity waveform was recorded on the ultrasound screen by another operator.

### Assessment of preload reserve

Maternal preload reserve was assessed using PLR as described previously [16]. The changes of SV (ΔSV) and CO (ΔCO) from baseline to 90 s after PLR (i.e., value after PLR − baseline value) were used to indicate the preload reserve. The percent change from baseline to PLR was calculated as: (value after PLR − baseline value)/baseline value × 100%. The information on the course and the outcome of pregnancy were obtained from the electronic medical records.

### Statistical analysis

All analyses were performed with IBM SPSS Statistics version 24. Bland–Altman analysis [26] was used to determine the agreement between preload reserve measured by ICG (ΔCO_ICG_ and ΔSV_ICG_) and echocardiography (ΔCO_echo_ and ΔSV_echo_). The bias and precision were calculated as mean difference and 1.96 SD of the mean difference, respectively [27]. Correlation between methods was assessed using Pearson’s coefficient. Continuous data are reported as mean ± SD.

### Ethical considerations

This study was approved by the Regional Committee for Medical and Health Research Ethics-North Norway (Ref.nr. 2015/575-2. Date of approval: 10.02.2010). Written informed consent was obtained from all participating women.

## Results

A total of 53 pregnant women were included in the final analysis. The baseline demographic, anthropometric, and clinical characteristics of the study population are presented in Table 1. A total of 31 women were in the second trimester and 22 were in the third trimester of pregnancy. Three women delivered preterm before 37 weeks; two vaginally at 36^+0^ and 35^+1^ weeks, respectively, due to spontaneous rupture of membranes and one at 32^+4^ weeks by a cesarean section because of placental abruption. These women were not excluded from analysis as they did not have any other complications during pregnancy, and their neonates were appropriate size for the gestational age at birth. The functional hemodynamic parameters measured by ICG and Doppler echocardiography are presented in Table 2. The preload reserve expressed as percent change in SV or CO from baseline to PLR was approximately 6% for Doppler echocardiography compared to 2% for ICG. ΔSV_ICG_ and ΔCO_ICG_ were slightly lower than ΔSV_echo_ and ΔCO_echo_, although the differences did not reach statistical significance (*p* = 0.053).

**Table 1:** Baseline characteristics of the study population (*n* = 53)

Parameter	Result
Maternal	
Age (years)	31 (20–39)
Gestational age at study (weeks)	23^6/7^ (17–41)
Weight (kg)	74 ± 14
Height (cm)	166 ± 6
BMI (kg/m^2^)	27.0 ± 4.4
MAP (mmHg)	80 ± 8
Fetal	
Gestational age at birth (weeks)	39^5/7^(32^4/7^–42^3/7^)
Birth weight (g)	3553 ± 507
Placental weight (g)^a^	615 ± 133
5-min Apgar score	10 (2–10)
Umbilical artery pH^b^	7.21 ± 0.10
Umbilical artery base excess (mmol/l)^b^	− 6.03 ± 3.81

Data are presented as median (range) or mean ± SD as appropriate

^a^1 missing values

^b^21 missing value

**Table 2:** Functional hemodynamic parameters measured by impedance cardiography and Doppler echocardiography

Parameter	Baseline	PLR	% Change	*p* value*
HR_ICG_ (bmp)	78 ± 12	78 ± 13	0.90 ± 9.65	0.669
HR_echo_ (bmp)	76 ± 12	76 ± 14	0.61 ± 9.72	0.752
SV_ICG_ (ml)	80 ± 18	81 ± 18	2.33 ± 12.87	0.407
SV_echo_ (ml)	71 ± 15	74 ± 17	5.72 ± 12.53	0.004
CO_ICG_ (l/min)	6.1 ± 1.4	6.2 ± 1.2	2.07 ± 11.69	0.623
CO_echo_ (l/min)	5.3 ± 1.0	5.5 ± 1.0	5.86 ± 12.43	0.007

% change is the difference between values obtained at baseline and 0.90 s after passive leg raising (PLR) calculated as: (measurement during PLR − measurement at baseline)/measurement at baseline × 100

Data are presented as mean ± SD

*Based on paired sample *t* test between baseline and PLR

**Fig. 2:**
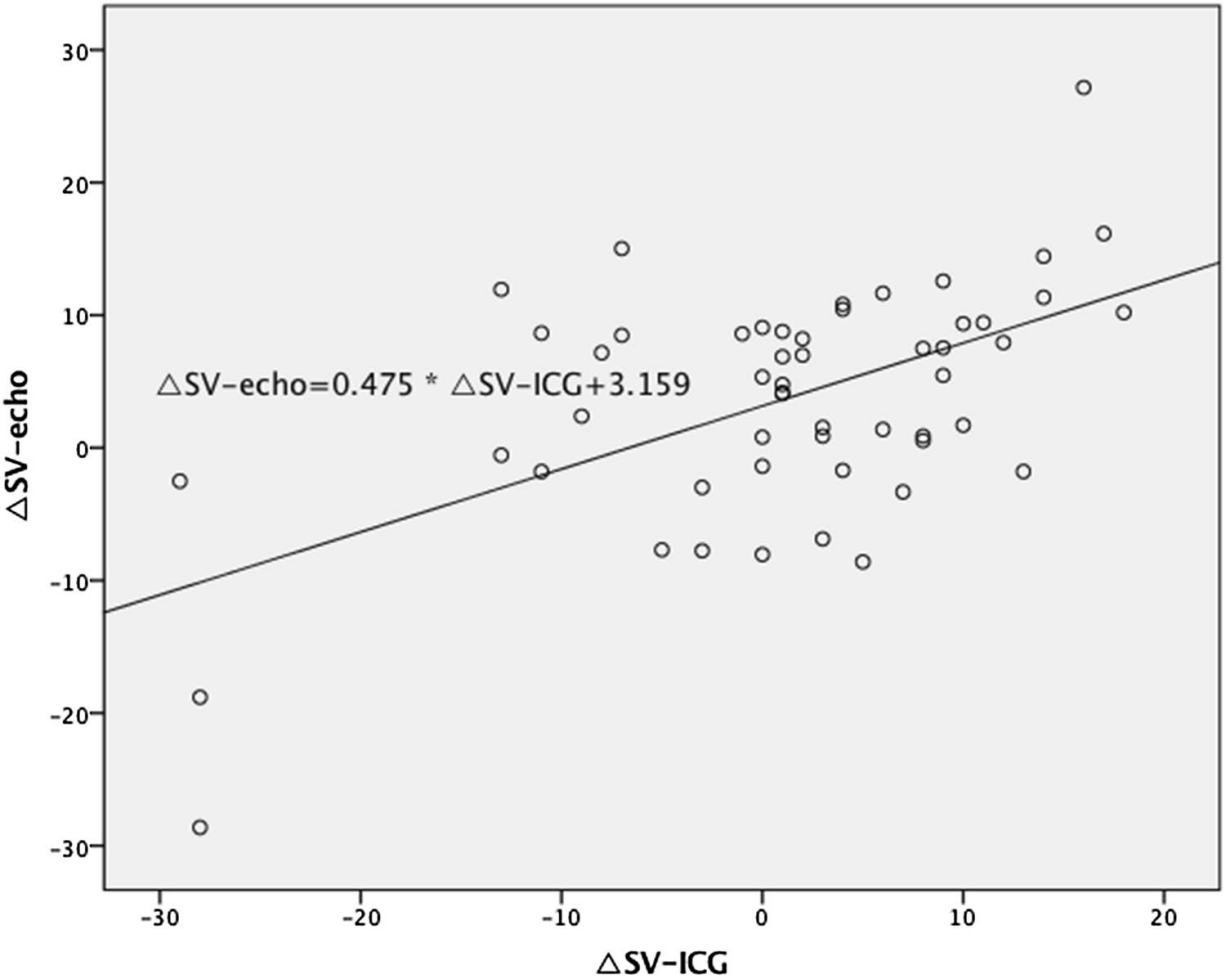
The correlation between the change in stroke volume from baseline to 90 s after passive leg raising measured by impedance cardiography (ΔSV_ICG_) and echocardiography (ΔSV_echo_)

**Fig. 3:**
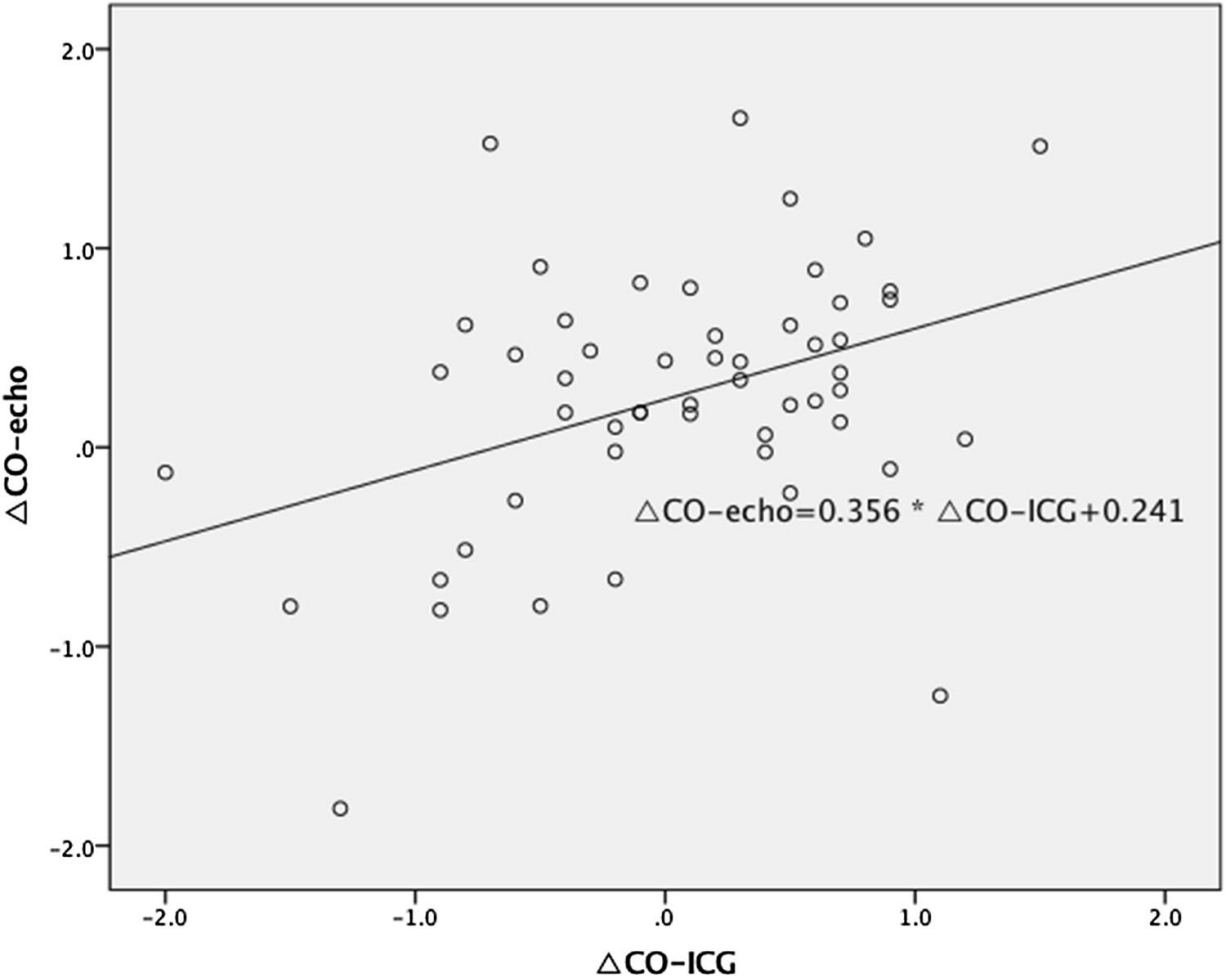
The correlation between the change in cardiac output from baseline to 90 s after passive leg raising measured by impedance cardiography (ΔCO_ICG_) and echocardiography (ΔCO_echo_)

**Fig. 4:**
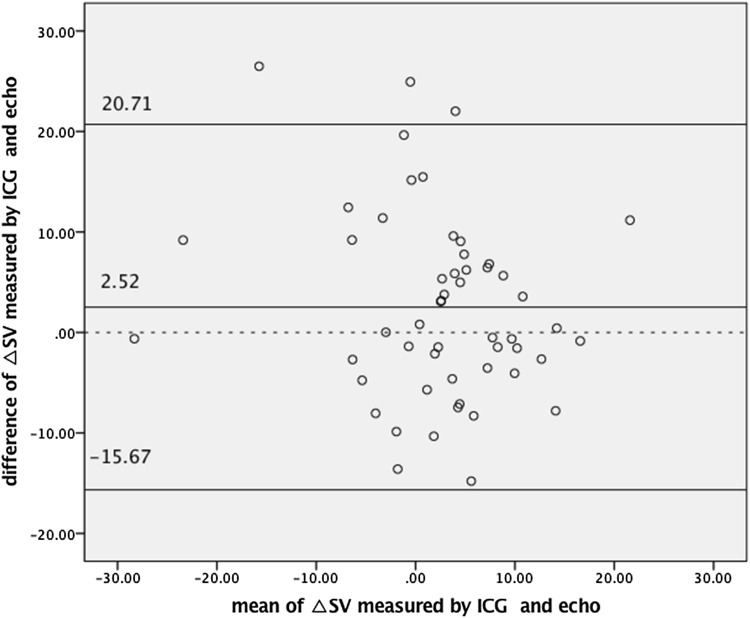
Bland–Altman plot depicting the agreement between the change in stroke volume from baseline to 90 s after passive leg raising measured by impedance cardiography (ΔSV_ICG_) (ml) and echocardiography (ΔSV_echo_)

**Fig. 5:**
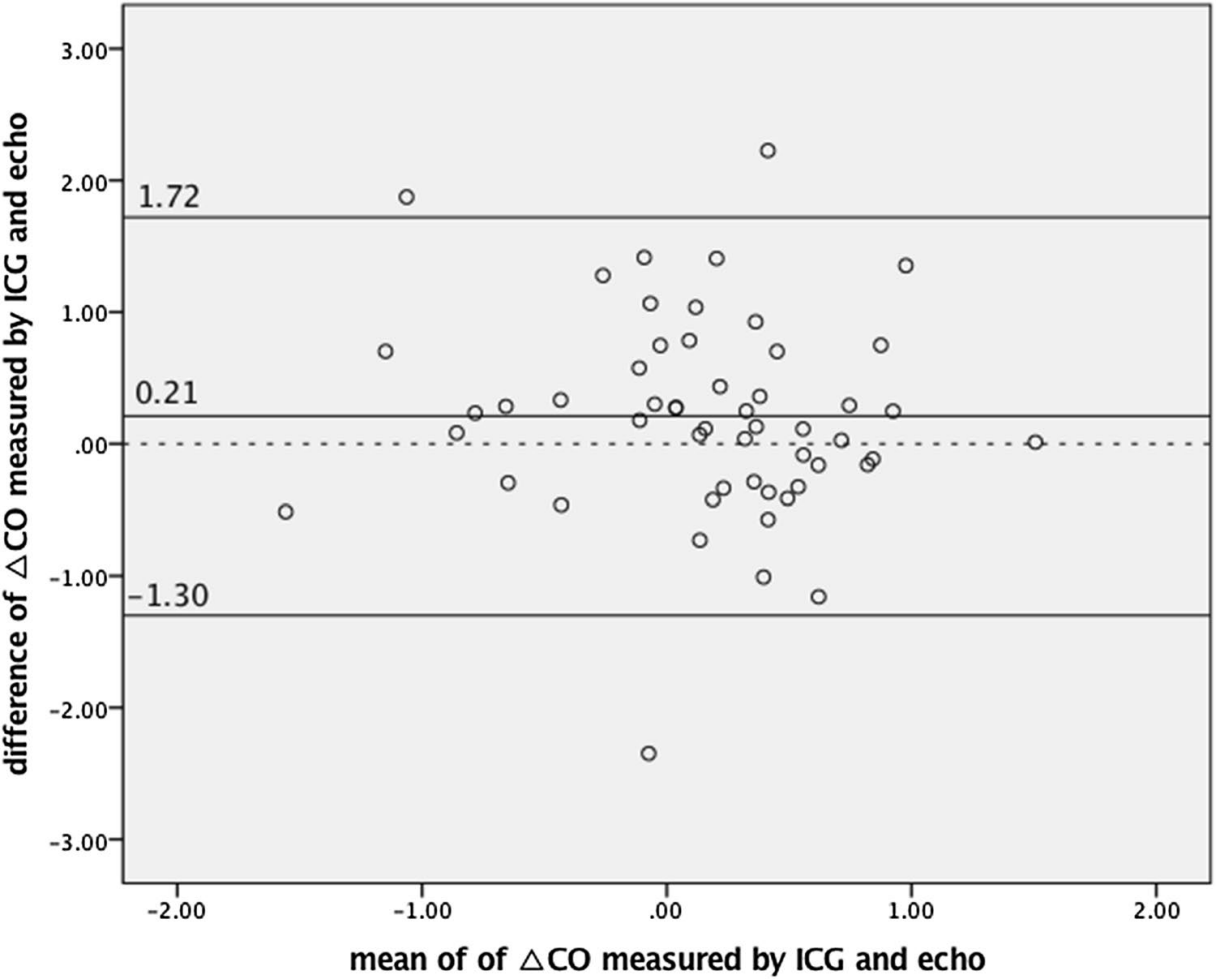
Bland–Altman plot depicting the agreement between the change in cardiac output from baseline to 90 s after passive leg raising measured by impedance cardiography (ΔCO_ICG_) (l/min) and echocardiography (ΔCO_echo_)

We found a statistically significant correlation between ΔSV_ICG_ and ΔSV_echo_ (*R* = 0.56, *p* < 0.0001) (Fig. 2) and between ΔCO_ICG_ and ΔCO_echo_ (*R* = 0.39, *p* = 0.004) (Fig. 3). The agreement between preload reserves measured by ICG and Doppler echocardiography is presented in Figs. 4 and 5. The mean difference (bias) for ΔSV was 2.52 ml, with a precision of 18.19 ml. The mean difference (bias) for ΔCO was 0.21 l/min, with a precision of 1.51 l/min.

## Discussion

Both ICG and Doppler echocardiography are widely used for the evaluation of heart function in the nonpregnant as well as pregnant populations. Both methods are useful as they are noninvasive, reproducible, and cost effective. However, the agreement between these two methods for measuring preload reserve during pregnancy remains still unclear. To our knowledge, this is the first study that reports the agreement between preload reserve measured by ICG and Doppler echocardiography during pregnancy.

The knowledge of preload reserve could be important in managing women with hypertensive pregnancy disorders, congenital heart disease, cardiac failure, massive obstetric hemorrhage and sepsis to guide management with drug therapy (such as anti-hypertensives, diuretics, and vasopressors), intravenous fluid therapy, and blood transfusion. The assessment of preload reserve has been shown to be useful in guiding fluid therapy in women with severe preeclampsia and oliguria [10]. However, the availability of a validated sensitive, accurate, reproducible, and user-friendly method of evaluating preload reserve is a prerequisite for it to be clinically applicable.

We found a reasonable agreement between preload reserves measured by two different noninvasive methods, i.e., ICG and echocardiography, with clinically acceptable bias of 2.52 ml for ΔSV and 0.21 l/min for ΔCO, and a statistically significant correlation between the ΔSV and ΔCO measured by Doppler and ICG techniques. The preload reserve expressed as percent change in SV or CO from baseline to PLR was higher when measured by Doppler echocardiography compared with that measured by ICG. Echocardiography is reported to have a precision of approximately 30% compared with gold standard invasive methods [27], which is considered clinically acceptable. ICG is simple, not operator dependent, and shown to detect small changes in SV related to postural change [28], but whether it accurately predicts preload reserve is not known. On the other hand, Doppler echocardiography has been reported to accurately predict preload reserve and fluid responsiveness in patients with severe preeclampsia [10]. Thus, it is important to consider the accuracy as well as other pros and contras of measurement techniques when interpreting the results of functional hemodynamic assessment during pregnancy. Although during pregnancy, there may be compression of the inferior vena cava by the gravid uterus and an increased abdominal pressure [29–31] that may alter hemodynamic response to PLR, elevated CO is adequately maintained in pregnancy during the postural challenge [32]. However, pathological processes, such as preeclampsia, maternal congenital or acquired heart disease, congestive cardiac failure, peripartum cardiomyopathy, severe anemia, hemorrhage, sepsis, and renal failure, might alter the preload reserve and fluid responsiveness during pregnancy. Therefore, the choice of a reliable noninvasive method to measure preload reserve is very important to accurately assess maternal functional hemodynamics.

One of the strengths of our study is that the ICG and Doppler echocardiographic measurements were performed by two single experienced operators each, eliminating inter-observer variability and allowing simultaneous acquisition of data using two different methods of evaluating preload reserve. However, there are some limitations of our study. First, the sample size of this study was not large enough to evaluate the differences in agreement between two methods in different gestational weeks. Second, we acknowledge that neither the Doppler echocardiography nor the ICG can be considered as a gold standard as they both have their own limitations in estimating SV and CO. However, the risk associated with invasive methods precludes their use in normal pregnancy. Third, since our study was cross sectional and we assessed participants on a single occasion, we did not account for the within-participant variability. This would be an important factor to consider if serial assessments’ preload reserve were performed during pregnancy. Longitudinal reference values for preload reserve measured by ICG are available for the second half of normal pregnancy [15], but not for the preload reserve measured by Doppler echocardiography. However, our results were within the normal range irrespective of the method used.

Since we did not include an invasive method that could be considered to be a gold standard in our comparison, it was not possible to identify which of the methods used are more accurate. However, the differences were small enough to be acceptable in clinical practice, and as there was a significant correlation between the preload reserve measured by the two techniques, any one of the two methods could be used to predict preload preserve in pregnancy. The considerable variation in agreement between the methods observed among the individual study participants may reflect the limitations of both techniques.

## Conclusion

There was a good agreement and a statistically significant correlation between ICG and Doppler echocardiography for measuring preload reserve suggesting that any technique could be used for this purpose. However, these methods should not be used interchangeably due to considerable variation in the agreement observed between individual subjects.
